# Task-related differences in network connectivity and dynamics in people with severe opioid use disorder compared with healthy controls

**DOI:** 10.1038/s41398-026-03845-6

**Published:** 2026-02-03

**Authors:** Danielle L. Kurtin, Katherine Herlinger, Alexandra Hayes, Lexi Hand, Leon Fonville, Raymond G. Hill, David J. Nutt, Anne R. Lingford-Hughes, Louise M. Paterson

**Affiliations:** 1 Division of Psychiatry, Faculty of Medicine, Imperial College London, London, UK; 2 Invicro London LLC, London, UK; 3 Department of Metabolism, Digestion and Reproduction, Faculty of Medicine, Imperial College London, London, UK

**Keywords:** Addiction, Neuroscience

## Abstract

One approach to addressing the immense unmet need for treatments of severe opioid use disorder (sOUD) is to understand more about associated changes in the brain’s reward circuitry. It has been shown that during reward anticipation in the Monetary Incentive Delay (MID) task, people with severe substance use disorder (SUD) show blunted responses in reward neural circuitry compared with healthy controls (HC). Conversely, drug-related cues result in heightened responses in the same neural reward circuitry in those with SUD compared with HC. However, it is unclear how such dysfunctional reward processing is related to neural correlates of other processes commonly dysregulated in addiction, such as attention and cognition. The aim of this work was to evaluate whether people with sOUD show different relationships between reward networks to networks that regulate cognition, attention, sensory processes, and more. Then, we evaluated whether there is a spatial relationship between differences in brain function and atlases of μ-opioid receptor (MOR) and dopamine D_2_ receptor (DRD2) availability. We collected fMRI data while people with sOUD receiving methadone (MD; n = 25) and HC (n = 22) completed the MID and cue reactivity tasks. We evaluated differences in functional connectivity (FC) and measures of brain state dynamics. Partial least squared (PLS) analysis computed the spatial relationship between FC metrics to MOR and D2DR availability. We found that MD participants generally exhibited weaker miFC compared to HC in both tasks except when comparing the difference in miFC during anticipation of monetary reward or drug related stimuli vs neutral stimuli. Contrasts between rewarding or drug-related to neutral stimuli showed MD participants had stronger miFC between reward/anti-reward networks to regions in the control network and default mode Network (DMN) in both tasks. Analysis of brain state dynamics showed the DMN was more prevalent in MD participants during the MID task. PLS analysis showed spatial autocorrelation between MOR and D2DR availability and connectivity metrics during the MID task. These findings reveal distinct patterns of neural network interactions in individuals with sOUD, characterized by generally reduced FC but enhanced connections between reward-related networks and cognitive control regions in response to either monetary or drug-related cues vs neutral cues. We observed spatial correspondence between receptor availability and altered connectivity and dynamics in MD vs HC. These results provide new insights into the neural basis of reward processing dysfunction in sOUD and may inform the development of targeted neuromodulation therapeutic approaches. Clinical trial registration: This study is not a clinical trial and therefore was not registered as a clinical trial. The study design and planned analytical approach for the primary analyses was pre-registered [1]. This paper consists of secondary analyses which were not primary considerations when designing the research study that collected the data.

## Introduction

Illicit opioid use contributes substantially to record-high drug-related mortality rates [2]. Limitations in current treatments for severe Opioid Use Disorder (sOUD) and illicit opioid use, such as cognitive-based interventions and opioid substitution therapy (OST), contribute to both record-high drug-related mortality rates [2], and relapse rates exceeding 60% within six months of treatment [3]. Altogether, there is an urgent need for more effective treatment strategies for sOUD.

One approach to informing therapeutic interventions is for sOUD to utilise insights from neuroimaging data. At least two reviews have highlighted the therapeutic potential of neuroimaging-informed noninvasive brain stimulation (NIBS) [4, 5]. For example, a clinical trial targeting low-intensity focused ultrasound stimulation (LIFU) at neuroimaging-informed markers of sOUD has shown therapeutic promise [6]. Eight people with sOUD received LIFU to the nucleus accumbens (NAc) while viewing drug cues. At the 30-day follow-up, only one had relapsed [6]. While caution should be exercised not to over-interpret the results of an ongoing trial, it is worth considering the factors that contributed to the clinical success of the stimulation. Successful stimulation targets are mechanistically grounded [7] with effects on brain function and clinically relevant behaviours that are reliable [8] and generalise across states/contexts [9]. Disrupted reward/antireward processing represents one of the most reliable biomarkers in sOUD [10], primarily affecting regions within the ventromedial Network (VMN), including the NAc, medial orbitofrontal cortex (mOFC), medial prefrontal cortex (mPFC), hippocampus, caudate, and amygdala. However, whether VMN dysfunction relates to other affected neural processes in sOUD, whether these relationships are context-dependent, and how they correspond to the spatial distribution of mechanistically relevant receptors remains unclear.

To address these gaps, this work aimed to characterise: (1) disrupted relationships among networks mediating attention, cognition, reward/antireward, and social/self-referential processing in sOUD; (2) whether functional disruptions generalize across contexts; and (3) spatial correspondence between altered brain function and receptor distributions mechanistically linked to sOUD development and maintenance.

We applied connectivity and dynamics analyses to fMRI data from the Neural Correlates of Reward and Emotion in opiate dependence (NCORE) study [1], which collected data from healthy controls (HCs) and methadone-dependent (MD) participants with sOUD during two tasks. The monetary incentive delay (MID) task evaluated neural responses to non-drug rewards, with reward anticipation periods as the condition of interest, when the processes disrupted by addiction are most strongly engaged [11, 12]. The cue reactivity task presented alternating blocks of drug-related and neutral stimuli, with drug-related blocks as the condition of interest [13, 14]. These tasks enabled assessment of whether connectivity and dynamics display corresponding task-dependent directionality, since they reliably elicit hyper- or hypo-activation of the VMN in sOUD versus HC participants.

Connectivity analyses capture the coordination between regions, offering insights how they interact as networks rather than in isolation [15]. We employed mutual information functional connectivity (miFC), an information-theoretic measure capturing both linear and nonlinear dependencies in interregional communication [16, 17], making it more sensitive than correlation-based measures [18, 19]. Additionally, we evaluated whether differences in connectivity between MD and HC participants differed during the whole task vs the conditions of interest, and during the condition of interest vs neutral conditions. This allowed us to assess whether the connectivity differences between groups were consistent and/or amplified during these critical task phases, thereby reinforcing the link between altered connectivity and the processing of natural rewards and/or drug-related cues in sOUD.

We hypothesised there would be task-dependent differences in MD vs HC miFC: during MID, MD participants would exhibit weaker VMN connectivity to default mode network (DMN), salience network (SN), and control network regions, reflecting weakened influence of natural rewards on self-referential, attentional, and cognitive processes [20–22]. We expect the miFC differences between HC and MD participants would be similar during both the entire MID task and the reward anticipation periods, reflecting the insensitivity to both positive and negatively valanced events [12], and reportedly weaker connectivity between people with SUD vs HC during both loss and win conditions [20–22]. During cue reactivity, we expect MD participants to show greater VMN-to-other-network connectivity reflecting enhanced incentive salience of drug cues [13, 14]. We expect these differences to be more prevalent during drug-related cues, due to the hyperactivity of VMN regions to drug-related stimuli in people with sOUD [13, 14] as compared to HC during the cue reactivity task, as well as observed greater connectivity in VMN regions in people with stimulant and alcohol use disorder vs HC during cue exposure [23, 24].

While miFC quantifies the relationship between two brain regions over time, metrics of brain state dynamics describe how networks are organised across time. Brain function is supported through the formation, maintenance, and dissolution of functional networks, and the inability to organise network dynamics over time has been associated with several disease states [25–27], We evaluated brain state dynamics by assigning each fMRI volume a network label and quantifying network prevalence across tasks [28]. We hypothesized MD participants would show lower VMN prevalence during MID (reflecting hypoactivity to non-drug rewards [11, 12]) but higher VMN prevalence during cue reactivity (reflecting hyperactivity to drug cues [13, 14]).

Finally, Partial Least Squares (PLS) analysis identified latent variables that capture the spatial relationship among the functional differences in MD vs HC and the receptor availability of two key neurotransmitters involved in the neurobiology of sOUD: μ-opioid receptor (MOR) and dopamine D_2_ receptor (D2DR). The maps of MOR and D2DR availability were sourced from a publicly available repository of positron emission tomography (PET) maps aggregated across many studies [29]. These publicly available maps enabled exploration as to whether the regional functional differences observed in the data related to the molecular properties of those regions, given their role in sOUD. While sOUD is associated with many disruptions in many (if not all) neurotransmitter and neuropeptide systems [5], we used MOR receptor availability maps because the use of illicit heroin and methadone in our MD participants operates on the MOR system [30]. It then follows that the spatial distribution of MOR receptor availability may relate to that of the regional differences in function between HC and MD participants. Similarly, there is a substantial body of literature implicating D2DR availability in the acquisition, development, and maintenance of addiction [5], as well as the receipt of naturally rewarding stimuli [31]. A region exhibiting a positive relationship between high MOR and/or D2DR availability and the different function in HC vs MD participants suggests that the effects of sOUD on MOR and/or D2DR receptor availability are predominantly conserved in areas with molecular properties related to the drug use. Conversely, weak spatial relationships between MOR and/or D2DR availability and differences in HC vs MD function suggests that the effects of heroin or methadone cascade beyond the areas with high receptor availability. Given our hypothesis that differences in miFC between HC and MD participants will predominantly be within the VMN, and that the VMN has high MOR and D2DR availability [32, 33], we hypothesise the PLS will identify a latent variable with a positive relationship between areas with high MOR and D2DR availability and differences in connectivity in both tasks.

## Methods

### Experimental design

Data from the NCORE project was used in this work. NCORE employed fMRI to characterise whether aprepitant (a neurokinin-1 receptor antagonist) modulated the neural correlates of reward processing, emotional processing, and cue reactivity in people with moderate-severe sOUD receiving methadone in comparison with HC. Only data from the placebo scanning session was used in the analyses described below. A detailed description of the study design can be found elsewhere [1], and a description of the MID and cue reactivity tasks can be found in Supplementary Materials, Task Description. An a-priori power calculation to determine sample size was conducted for the NCORE study, and a description of study power as applied to this work can be found in Supplementary Materials, Power Calculation.

### Participants

MD participants (n = 29) were recruited from community-based NHS and/or voluntary sector run drug and alcohol services based in London and surrounding areas. Potential participants were identified by via investigator led caseload screening, direct keyworker/clinician referral, or advertisement at services. Healthy volunteers (n = 22) were recruited via advertisement in press, on social media, volunteer databases or poster advertisements.

Inclusion criteria for all participants were as follows: (i) aged over 18 years; (ii) males or females. Exclusion criteria for all participants included: (i) intoxication at any of the screening or study visits; (ii) positive drug (except for cannabis) and alcohol screens at any of the screening or study visits; (iii) the use of regular relapse prevention medications; (iv) any physical or mental health issues that may affect study safety or integrity; (v) any MRI contraindication.

Main inclusion criteria for MD individuals included: (i) DSM-5 diagnosis of current, severe sOUD; (ii) currently treated with methadone substitution therapy (<60mgs/day) and able to maintain the same dose across two scanning sessions separated by at least five days. Main exclusion criteria for MD participants included: (i) current alcohol or substance use disorder apart from opioid or nicotine; (ii) current severe DSM-5 mental health disorder; (iii) regular on-top use of heroin, opiates or other illicit substances except cannabis.

Main exclusion criteria for HC included: (i) current or history of substance or alcohol use disorder apart from nicotine; (ii) current or history of pathological gambling; (iii) current DSM-5 psychiatric disorder; (iv) current regular use of psychotropic medication that cannot be paused for the duration of the study.

The study was approved by the West London & GTAC ethics committee (REC:19/LO/0971) and performed in accordance with the principles of the Declaration of Helsinki. All participants provided written informed consent.

### MRI acquisition and preprocessing

The Supplementary Materials contain sections on MRI Acquisition and Quality Assessment metrics (e.g., motion artefact and temporal signal to noise ratio (tSNR)). Preprocessing was conducted using the fMRI Expert Analysis Tool (FEAT, Version 6.00) from the FMRIB Software Library (FSL) [34, 35]. Preprocessing consisted of interleaved slice-time correction, brain extraction using FSL’s brain extraction tool (BET), motion correction in 6 degrees of freedom with FSL’s MCFLIRT, and spatial smoothing with a 5 mm at full-width at half maximum Gaussian kernel filter. Framewise displacement was computed as metric of motion artefact. Subject-level independent component analysis (ICA) was conducted with ICA AROMA algorithm [36], which classified each of the data-driven number of components as either signal or noise. Components identified as noise were regressed out using FSL’s *regfilt* function.

### Mutual information functional connectivity (miFC)

We used mutual information as a nonparametric assessment of dependence between the timeseries of two brain regions [17, 37–39]. MiFC is an information theoretic metric that quantifies “connectivity” as the extent to which observing the state of one region reduces the entropy (i.e., uncertainty) of another [17]. While correlation-based connectivity quantifies linear or monotonic associations, miFC captures both linear and nonlinear dependencies in the infra-slow oscillations of fMRI timeseries [17]. MiFC has been shown to be more sensitive than correlation-based metrics at detecting interregional relationships in healthy controls [18], and better at capturing behaviourally relevant measures of brain function in clinical populations [19]. For a more complete description of the advantages of miFC and its relevance to brain function, we refer readers to our previous work implementing miFC on task data in healthy humans [16].

**Fig. 1:**
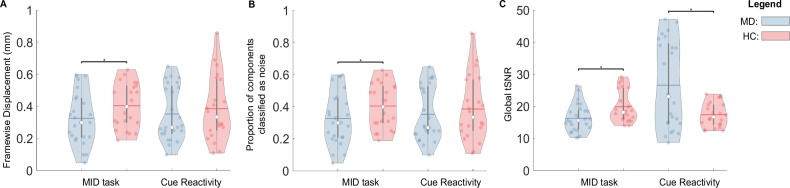
Violin plots of three quality assessment metrics. **A** the mean FD per subject, (**B**) the proportion of components classified as noise, and (**C**) the global tSNR. The top and bottom edges of the boxes represent the 25th and 75th percentiles, with the mean shown by the horizontal line and the median by the white dot. Extension of the whiskers indicates 1.5 the interquartile range. Individual datapoints are shown, and a kernel density estimate of the data provides the edges to the violin plot. * indicates a significant difference with p < 0.05.

Regions were defined by transforming the 200-parcel, 17-network Shaefer atlas [40, 41] to subject space using ANTs. Anatomical parcellation of subcortical regions was conducted using Freesurfer’s *recon_all* function [42]. The remaining steps to compute pairwise miFC were conducted in line with our previous work [16]. Timeseries were extracted from each region and z-scored to centre mean 0 and ±1 standard deviation (std). Algorithms to compute pairwise miFC [43] generated a symmetric 214-by-214 miFC matrix (Fig. 1Ai).

To evaluate whether differences in miFC between HC and MD participants are driven by the condition of interest in each task, we created a “task timeseries” for each participant, and computed pairwise miFC during the volumes obtained during the condition of interest (Fig. 1Aii). For example, the condition of interest in the cue reactivity task are the blocks of drug cues. To compute miFC during blocks of drug cues, we initially create a “task timeseries” as an array of “0”’s, one for every second in the acquisition. Then, periods in which drug cues are presented are coded as “1”, similar to the “boxcar regressors” used in whole brain GLM models of voxelwise activity. Periods of drug cue presentation are obtained from the explanatory variable, which contains at what time and for how long drug cues were presented, in seconds. This same procedure is applied during the MID task, where the condition of interest (i.e., periods of reward anticipation) are coded as “1”.

The task timeseries was convolved with a canonical haemodynamic response function using SPM, thus aligning the experimental time with the BOLD signal. Then, the task timeseries is downsampled to align with the TR at which fMRI volumes were collected. We then extract and concatenate volumes from regional timeseries where the task timeseries is coded as “1”. The remaining miFC computation and statistical analysis was conducted in line with that performed on the entire task timeseries.

We also computed the differences in miFC between HC and MD during contrasts of the conditions of interest to neutral conditions (e.g., drug cues>neutral cues, reward anticipation > neutral or loss anticipation).

### Multivariate mapping between μ-Opioid Receptor and DRD2 availability and differences in brain function between MD and HC participants

In line with the analysis conducted by Hansen and colleagues [29], we employed a PLS analysis to assess the extent to which regional receptor availability (i.e., molecular predictors) related to connectivity-based differences between MD and HC (i.e., functional responses). In this PLS, we had two molecular predictors (regional MOR and D2DR receptor availability) and the following four functional responses: MID eigenvector centrality_MD-HC_, cue reactivity eigenvector centrality_MD-HC_, MID degree_MD-HC_, and cue reactivity degree_MD-HC_).

The regional receptor availability was derived from normative PET atlas of 19 different receptor images created by a study that collated PET images from 26 studies [29]. The MOR availability image was created using data from two studies. However, we only used data from one study by Kantonen et al., due to its large sample size (n = 204) [44], and the other, smaller study included participants from the study by Kantonen in their cohort, rendering their PET images non-independent [45]. In line with Hansen et al., an average DRD2 image was created from the mean of three studies [46–48].

Regional receptor availability from the same 200-region Schaefer atlas used in the connectivity analysis was provided with the atlas images. To obtain the receptor availability for the subcortical regions included in this work, regions from the Melbourne subcortical atlas were used to extract receptor availability from the volumetric PET images [49]. The Scale 1 parcellation of the subcortical atlas splits the anterior and posterior thalamus [49]; therefore, the anterior and posterior thalamus were combined into a single region, to harmonize the parcellation scheme employed for the connectivity analysis.

Then, as in Hansen et al., the receptor densities were z-scored across all regions to centre mean 0 with ±1 std. To increase the interpretability of computing the pairwise sum of receptor densities, the z-scored receptor densities were rescaled between 0 and 1.

As aforementioned, functional responses consist of regional connectivity-based differences between MD and HD participants. Degree was computed as the total number of edges with a significantly different miFC (regardless of direction) between MD and HC participants. Since degree captured the number of differences in a region, we computed Eigenvector Centrality (EC) to assess the extent to which those differences the importance of a region in mediating whole-brain communication [50]. Eigenvector centrality_MD-HC_ was computed for each region by subtracting the mean eigenvector centrality among MD participants from the mean of HC.

The PLS computes latent variables that characterise the spatial relationship between molecular properties and functional responses. The variable importance in projection (VIP) score is a cumulative measure that represents the influence of a variable on the model across all components. VIP scores are calculated as a weighted sum of squares of the PLS weights while considering the amount of explained variance in response variables in each dimension. A VIP > 1.0 indicates a variable that is important in the model.

Latent variables are composed of scores for each region, which denotes the magnitude of its contribution to the latent variable. By averaging the scores among regions within each functional network, we derive a measure of the extent to which each network’s molecular and functional properties contribute to the latent variable.

### Defining brain states

Regional BOLD signal timeseries were demeaned, band-pass filtered by a 2^nd^ order Butterworth filter (passband of 0.02–0.1 Hz), then Hilbert transformed to an analytic signal as in Eq. 1:

1 $$X\left(t\right)=A\left(t\right)\cos \left(\theta \left(t\right)\right),$$

where *A(t)* is the instantaneous amplitude, and *θ(t)* is the instantaneous phase. The phase angle was used to compute a FC matrix as in Eq. 2:

2 $${FC}\left(n,p,t\right)=\cos \left(\theta \left(n,t\right)\right)-\theta (p,t))$$

where *FC(n,p,t)* contains the phase coherence between brain areas *n* and *p* at time *t*. *FC(n,p,t)* was masked by binary matrices representing each of the eight canonical functional networks [41] and the VMN [4]. The mean coherence across all regions included in each of the template networks was computed from the upper triangle of *FC(n,p,t)* with the diagonal omitted. Timepoint *t* was assigned the network with highest average coherence, generating a state timeseries (Fig. 1C).

To ensure dynamical FC results are not a byproduct of this method developed in-house (HomeBrew State Dynamics (HBSD)), we also computed brain states and state timeseries using a conventional dynamical FC analysis, Leading Eigenvector Dynamic Analysis (LEiDA) [28, 51]. Methods for LEiDA and its comparison to HBSD are provided in Supplementary Materials, Leading Eigenvector Dynamic Analyses: Methods.

Supplementary Materials Leading Eigenvector Dynamic Analysis: Results, and Supplementary Materials Tables 1–6, shows the extent to which the effect of group on metrics of brain state dynamics replicated those presented in the Results section.

### Metrics of brain state dynamics

Metrics of brain state dynamics were computed for the entire task. The lifetime and probability of each state’s occurrence, and three information theoretic metrics – Lempel Ziv complexity (LZC), block decomposition method of complexity (BDMC), and transition entropy – were computed as previously reported in our work [28] and others [51, 52]. The probability and lifetime of a state’s occurrence captures the dominance of a particular state across a time. State probability of occurrence is computed as the sum of the number of volumes assigned that state’s label divided over the total number of volumes. State lifetime is computed as the number of volumes assigned that state’s label multiplied by the TR.

While state lifetime and probability capture dynamical aspects of each state, LZC, BDMC, and transition entropy capture the diversity and predictability of how of *all* states are organised over time. Applied to state timeseries, LZC estimates statistical complexity as the number of unique patterns in a state timeseries [53]. BDMC is a measure of algorithmic complexity that works by computing the number of algorithms needed to generate a given sequence (e.g., state timeseries) [54, 55]. Further comparison on the merits of BDMC vs LZC applied to state timeseries, or their application to task fMRI data, can be found in our previous work [28]. LZC and BDMC were computed on 4-bit binarized state timeseries via an LZC algorithm, LZ76 [53] and BDMC algorithms [54, 56, 57], respectively.

Transition entropy, also known as block entropy, can be considered an information theoretic version of the direction transitions commonly assessed in dynamical analyses [51, 52]. Transition entropy for 0^th^-4^th^ order transitions was derived by first computing an N^th^ order (where N = 0–4) Markov Model representation of the sequence of brain states per person per condition, yielding a probability *p* for each type of transition present. The entropy (*H*) for each order of transition is then computed using Eq. 3:

3 $$H=-\sum (p.* lo{g}_{2}p)$$

### Statistical analyses

All analyses were run in MATLAB2023a. Permutation tests evaluated the main effect of group on miFC and dynamical metrics [58]. Significance was determined as p < 0.05 after max-T familywise correction for multiple comparisons. Among all edges with significantly different miFC between HC and MD participants for each task, the proportion of edges with significantly stronger or weaker miFC in MD vs HC participants was determined by counting the number of edges either positive or negative T-stats.

Spearman’s correlations assessed the strength of relationship between molecular predictors and functional responses to the latent variable in the PLS analysis. Spin tests were employed to ensure significant mappings between the molecular predictors and functional responses were not driven by spatial correlation [59]. A permutation map (i.e., shuffled ordering of regions) was created by using the *rotate_parcellation* function by Váša and colleagues [60], which rotates the coordinates of each region’s centre of gravity while preserving hemispheric symmetry. Then, the PLS analysis was run for 10,000 iterations, with each iteration shuffling the rows of the functional responses using the permutation maps. Significance of empirical tests was determined as a two-tailed t-test by comparing the empirical test statistics to the null distribution generated by permuted molecular predictor scores and the percent variance explained for each latent variable.

## Results

### Demographics, clinical information, image quality assessment

For HC and MD participants, 22 and 29 participants met the inclusion criteria and completed placebo scanning sessions, respectively. However, due to excessive motion (>20% of volumes in either task showed a FD > 0.9), 4 MD participants were excluded. Therefore, the final group size is n = 22 HC and n = 25 MD participants. The mean FD across participants included in this study was 0.18 (std = 0.08) and 0.22 (std = 0.14) for the MID and cue reactivity task, respectively. Permutation tests showed FD was significantly greater in HC compared to MD in the MID Task (p = 0.02, T-stat = 0.04, D = 0.58) (Fig. 1A). There was no significant group difference in FD in the cue reactivity task (p = 0.20, T-stat = 0.03, D = 0.25) (Fig. 1A).

The average proportion of components ICA AROMA classified as noise across participants was 36.34% (std = 16.68) and 36.89% (std = 18.20) for the MID and cue reactivity task, respectively. Permutation tests showed there were significantly more components identified as noise in the HC participants as compared to the MD participants during the MID task (p = 0.03, T-stat = −0.08, D = 0.52) (Fig. 1B). There was no effect of group on the number of components for the cue reactivity task (p = 0.23, T-stat = −0.03, D = 0.18) (Fig. 1B).

The global temporal signal to noise ratio (tSNR) was computed to assess the contribution of noise to the data. Permutation tests showed MD participants had significantly lower global tSNR than HC participants during the MID task (p = 0.01, T-stat = −3.57, D = 0.77) (Fig. 1C) and significantly higher global tSNR than HC during the cue reactivity task (p < 0.001, T-stat = 9.25, D = 0.94) (Fig. 1C). The mean global tSNR for each group in both tasks is above 15, and therefore sufficiently powered to detect minimum effects/changes in the magnitude of the BOLD signal of 1–5% [61], and aligns with the average tSNR of task-based studies [62].

Among participants included in the study, demographics for age, sex, and race were matched between MD and HC participants (Supplementary Materials Table 7). The number of participants with smoking status or current psychiatric diagnosis was significantly higher in MD vs HC participants, and the years of education was significantly lower in MD vs HC participants (Supplementary Materials, Table 7).

### Differences in miFC between HC and MD participants during the MID and cue reactivity tasks

The differences in miFC between HC and MD participants during each task are presented in two sections: first, significant differences in miFC between participant groups for the entire task; second, significant differences in miFC between participant groups for the condition of interest.

#### MID Task

##### Entire MID task

**Fig. 2:**
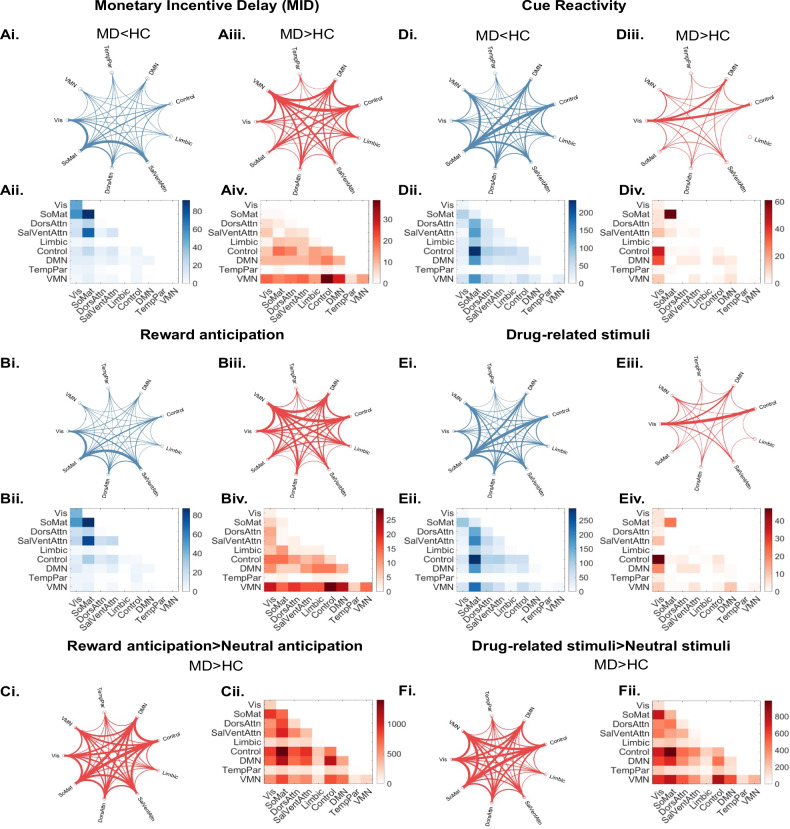
Differences in between-network connectivity in HC compared with MD participants during both the MID and cue reactivity tasks. There were significant differences in miFC between HC and MD participants during (**A**) the entire MID task and (**B**) it’s condition of interest, reward anticipation periods, and (**C**) the contrast between the reward vs neutral anticipation periods. There were also significant differences in miFC between groups during the (**D**) cue reactivity task, (**E**) it’s condition of interest, blocks of drug-related cues, and (**F**) the contrast between drug-related cues vs neutral cues. **Ai, Aiii, Bi, Biii, Ci, Ciii, Di, Diii, Ei, Fi** Circle plots showing edges that were significantly higher in MD participants vs HC (red lines), and vice versa (blue lines). Regions with differences in miFC grouped by functional network. The thickness of the lines between networks is proportional to the degree, or number of significant edges between the networks. **Aii, Aiv, Bii, Biv, Cii, Civ, Dii, Div, Eii, Fii** Connectivity matrices showing edges that were significantly higher in MD participants vs HC (red), and vice versa (blue). Connectivity matrices can show intra-network degree, whereas circle plots convey inter-network degree. Colourbars indicate the range of the degree. Vis visual, SoMat somatomotor, DorsAttn dorsal Attention, Sal/Vet salience/ventral attention, TempPar temporal parietal.

Permutation tests showed there were 921 edges with significantly different miFC between HC and MD participants (Supp Mat 8). Slightly more than half of MD participants had edges with significantly weaker miFC compared with HC (57.33 vs 42.67%). Edges with significantly stronger miFC in HC compared to MD participants tended to include regions in the somatomotor (38%), visual (21%), and salience/ventral attention (15%) networks (Fig 2Ai-ii). Edges with stronger miFC in MD vs HC participants were most often contained regions in the VMN (23%), followed by the control network (18%) and DMN (15%) (Fig 2Aiii-Aiv). Condition of interest: reward anticipation periods

While there were fewer edges with significantly different miFC between HC and MD participants during the reward anticipation periods than the entire task (879 vs 921), the networks in which the edges were concentrated were similar to those observed during the entire MID task (Supp Mat 9). The split between edges with significantly weaker (60.98%) or stronger (39.02%) miFC in MD participants compared with HC was similar to the split in the entire MID task.

As during the entire MID task, edges with significantly stronger miFC in HC as compared to MD participants remained predominantly within somatomotor (36%), visual (20%), or salience/ventral attention (18%) networks (Fig 2Bi-ii). Similarly, edges with significantly stronger miFC in MD vs HC participants tended to contain regions in the VMN (25%), control network (15%), and DMN (14%) (Fig. 2B).

### Condition of interest: reward anticipation > neutral anticipation

Permutation tests showed there were 22,512 edges with significantly different miFC between HC and MD participants (Supp Mat 10). All edges with significantly different reward anticipation > neutral anticipation miFC were stronger in MD compared with HC. Edges with significantly stronger miFC in MD compared to HC participants tended to include regions in the somatomotor network (18%), control network (17%), and DMN (14%) (Fig 2Ci-ii).

### Cue reactivity task

#### Entire cue reactivity task

Permutation tests showed there were 2206 edges with significantly different miFC between HC and MD participants (Supp Mat 11). There were more edges with significantly stronger miFC in HC participants (88.71%) than MD participants (11.29%) (Fig 2Di-Dii). Edges with significantly stronger miFC in HC participants were often in the somatomotor (25%), control (17%), or dorsal attention (13%) networks. In contrast, edges that had significantly stronger miFC in MD participants tended to include regions from either the somatomotor (28%), visual (26%), DMN (14%), or control (13%) networks (Fig 2Diii-Div).

### Condition of interest: cue blocks with drug-related stimuli

There was a greater number of edges with significantly different miFC between HC vs MD during the drug-related cue blocks (2679 edges) as compared to the entire cue reactivity task (2206 edges) (Supp Mat 12). As in the results from the entire cue reactivity task, there were more edges with significantly stronger miFC in HC participants (93.50%) than MD participants (6.50%). Edges with significantly stronger miFC in HC vs MD participants were often in the somatomotor (26%), control (17%), dorsal attention (12%), and salience/ventral attention networks (12%) (Fig 2Ei-Eii). Most edges with significantly stronger miFC in MD participants included a region in the visual (31%), control (20%), somatomotor (15%), or DMN (13%) networks (Fig 2Eiii-Eiv).

### Condition of interest: cue blocks with drug-related stimuli > neutral stimuli

Permutation tests showed there were 14,636 edges with significantly different miFC between HC and MD participants (Supp Mat 13). All MD participants had significantly stronger cue > neutral miFC compared with HC. Edges with stronger miFC in MD vs HC participants were most often contained regions in the Somatomotor network (17%), followed by the VMN and Control networks (17%).

#### Summary of differences in miFC between HC and MD participants across the MID and cue reactivity tasks

Across the MID and cue reactivity tasks MD participants showed similar patterns of disrupted miFC as compared to HC. Across both tasks and the condition of interest MD participants tended to have weaker miFC than HC, and the networks with the most regions contributing to edges with significant group differences in miFC during the whole task mirrored those in the conditions of interest.

In both the MID and the cue reactivity tasks we observed that regions with stronger miFC in MD vs HC participants tended to include regions in the DMN and control networks, and edges with stronger miFC in HC vs MD participants tended to include regions in the somatomotor network. One notable difference between tasks was that the VMN contained the most edges with significantly stronger miFC in MD vs HC during the MID, but not cue reactivity, task.

Interestingly, the contrast between the condition of interest vs neutral condition is similar across tasks, but substantially different from the task as a whole or just the condition of interest. For both tasks there was an order of magnitude more edges with significant group differences; moreover, all showed significantly stronger miFC during the condition of interest vs neutral condition in MD vs HC. While results were concentrated between the control and somatomotor networks, both tasks also showed significant differences in DMN, VMN, visual, and attention networks.

### Relationship between molecular properties and between-group differences in miFC

**Fig. 3:**
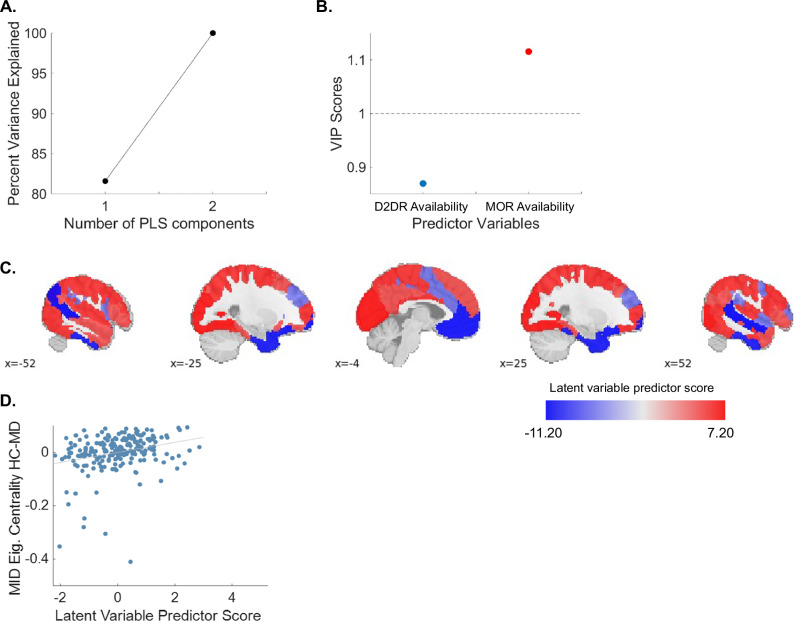
Partial least squared analysis showed relationships between receptor availability and differences in miFC between MD and HC participants. **A** Percent variance of the relationship between molecular predictors and functional responses by each latent variable. **B** The VIP score for each molecular predictor. **C** Spatial relationships between the latent variable predictor score and cortical regions. **D** Scatter plot of the functional response MID Eigenvector Centrality_HC-MD_ with the predictor score for the first latent variable.

PLS analysis computed latent variables that represent extent to which molecular predictors (i.e., availability of D2DR and MOR on HC, group-average maps) related to differences in brain function between HC and MD participants (i.e., the difference between HC and MD participants in MID and cue reactivity regional degree and eigenvector centrality). The first latent variable from the PLS captured 82% of the covariance in the relationship between the spatial distribution of receptors and functional differences between HC and MD participants. The first latent variable did not survive spin tests determining whether the results were not driven by spatial autocorrelation (p_spin_ > 0.30) (Fig. 3A). Out of the two molecular predictors, MOR receptor availability was an important contributor to the first latent variable (VIP = 1.12) (Fig. 3B). By averaging the regional molecular predictor score in each network, we found the VMN captures the maximal shared variance between receptor densities and functional responses (Fig. 3C).

Though the relationship captured by the primary latent variable may be influenced by spatial autocorrelation, it remains worth investigating whether any of the functional responses are associated with regional predictors and their spatial organisation. We assessed the extent to which the molecular regional predictor scores from the first latent variable related to each functional response. There was a significant relationship between predictor score of the primary latent variable and eigenvector centrality during the MID task (rho=0.30, p = 6.38e-6, p_spin_ = 4.00e-4) (Fig. 3D). There was no significant relationship between the predictor score of the primary latent variable and the eigenvector centrality during the cue reactivity task (rho = −0.11, p = 12, p_spin_ = 0.12), nor the difference in degree between HC and MD during the MID (rho = 0.06, p = 0.38, p_spin_ = 0.41) or cue reactivity tasks (rho = 0.13, p = 0.07, p_spin_ = 0.08).

### Brain state dynamics

**Fig. 4:**
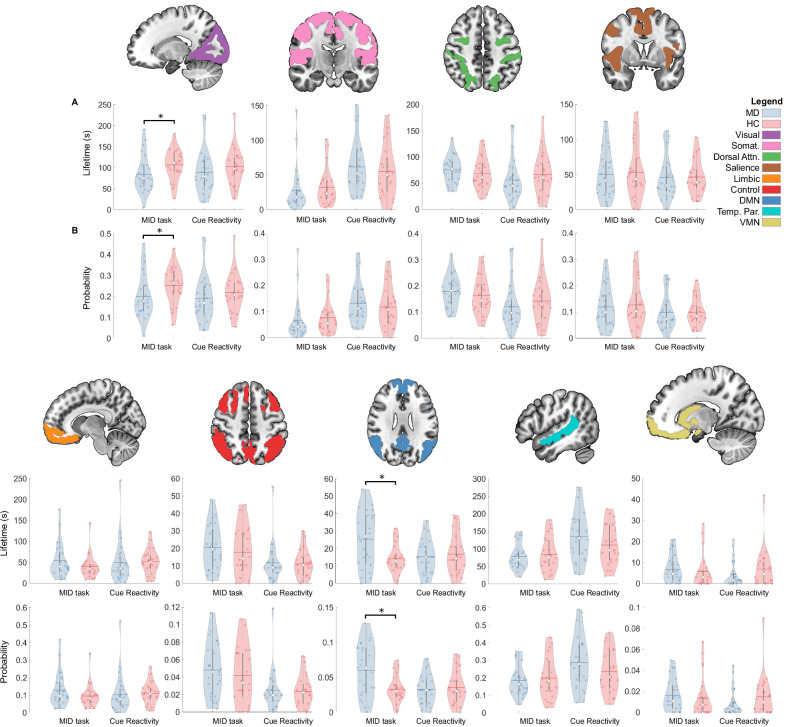
Differences in brain network dynamics in MD participants compared with HC. Violin plots of the state (**A**) lifetime and (**B**) probability for MD and HC participants in the MID and cue reactivity task. The top and bottom edges of the boxes represent the 25th and 75th percentiles, with the mean shown by the horizontal line and the median by the white dot. Extension of the whiskers indicates 1.5 the interquartile range. Individual datapoints are shown, and a kernel density estimate of the data provides the edges to the violin plot. Somat somatomotor, Attn attention, Temp. Par temporal parietal.

Two networks showed significant differences in standard metrics of brain state dynamics between MD vs HC participants during the MID task (Supp Mat 14). The lifetime (p = 0.03, T-stat = −22.37, D = 0.57) and probability of occurrence (p = 0.03, T-stat = −0.05, D = 0.57) for the visual network was significantly lower in MD vs HC participants (Fig. 4). The lifetime (p < 0.001, T-stat = 11.33, D = 0.91) and probability of occurrence (p = 0.001, T-stat = −0.03, D = 0.91) for the DMN was significantly greater in MD vs HC participants (Fig. 4).

**Fig. 5:**
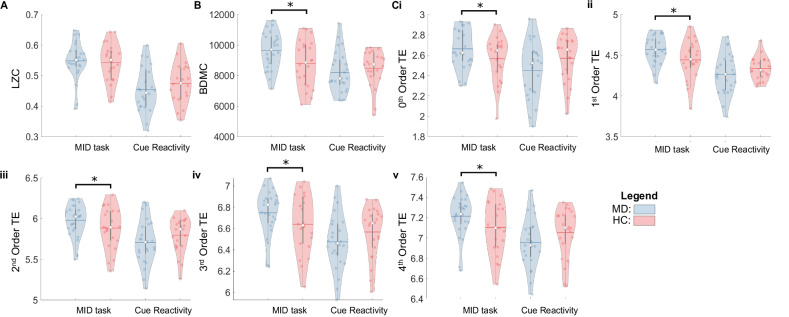
The complexity of brain state dynamics is altered in MD participants vs HC. Violin plots of the (**A**) LZC, (**B**) BDMC, (**Ci-v**) and 0–4^th^ order transition entropy (TE) for MD and HC participants in the MID and cue reactivity task. The top and bottom edges of the boxes represent the 25th and 75th percentiles, with the mean shown by the horizontal line and the median by the white dot. Extension of the whiskers indicates 1.5 the interquartile range. Individual datapoints are shown, and a kernel density estimate of the data provides the edges to the violin plot.

There were significant differences in complexity metrics of brain state dynamics between MD vs HC participants during the MID task (Supp Mat 15). MD participants had significantly greater BDMC and 0–4^th^ order transition entropy than HC (Fig. 5B-Ci-v). There was no significant difference in LZC between MD and HC participants during the MID task (Fig. 5A).

There were no significant group differences in metrics of brain state dynamics during the cue reactivity task (Figs. 4, 5, Supp Mat 14–15).

## Discussion

Our aim was to identify differences in brain function between MD and HC participants and evaluate whether differences were task dependent. Differences in brain function were captured through miFC and brain state dynamics, and a PLS assessed the spatial relationship between functional disruptions in MD participants to MOR and D2DR availability. These analyses extend the foundation of work characterising the differences in the magnitude of the BOLD signal in the VMN (i.e., reward- and anti-reward regions) between HC and SUD participants during both the MID and cue reactivity tasks [3, 14, 63–67]. The reliable hyperactivation of the VMN in sOUD during drug cue presentation [3, 14, 65, 68] has been deployed as a neuroimaging-informed target for neuromodulatory interventions [69]. Our multi-task, multimodal approach sought to identify additional biomarkers of sOUD, in hopes of contributing to a preliminary foundation of neuroimaging-informed targets for neuromodulation [4, 69].

Our study revealed unexpected patterns in miFC among brain networks in MD vs HC participants. The MD group showed stronger miFC between the VMN to the DMN and control networks. In contrast, HC participants exhibited stronger miFC within and between their visual, somatomotor, and salience/ventral attention networks. While we expected VMN engagement during the MID task due to its role in reward processing, anticipation, and outcome [70, 71], we had hypothesised that MD participants would show weaker miFC than HC participants. This hypothesis was based on previous research showing reduced VMN activity during reward anticipation in SUD [67], though findings about VMN engagement during reward anticipation and outcomes in SUD have been mixed [11, 66].

These findings can be better understood by examining how different networks relate to the distinct phases of trials in the MID task. Trials in the MID task begin with participants seeing whether they can win money, lose money, or neither. This initial phase likely engages the VMN, DMN, and control networks to process the potential value and motivate performance [70]. Next, participants wait to press a button when a target appears, requiring focused visual attention and motor readiness. This phase primarily involves sensory and attention networks [72]. Finally, participants wait, and then are presented with, their performance outcome, typically engaging reward circuitry—the focus of most MID research [66, 67, 70].

The network connectivity patterns suggest different task strategies between groups. HC participants showed stronger miFC in networks supporting the target-response phase (visual, somatomotor, and salience/ventral attention networks). This aligns with research showing HC had higher FC in visual and salience/ventral attention networks as compared to stimulus dependent participants during the MID task [73]. Similar patterns have been observed in studies of people with cocaine, opioid, or polysubstance use disorders [22]. Conversely, MD participants showed stronger connectivity among networks involved in reward processing, goal visualisation, and motivation (VMN, DMN, and control networks), especially when contrasting miFC during reward vs neutral anticipation. The substantial number of edges with significantly stronger miFC during reward vs neutral anticipation in MD vs HC participants suggests that that the reward- and loss-encoding role of the VMN formed a key component of the MD participants task performance strategy mediated by the DMN and control networks. This was supported by two factors. First, the consistent results across the whole task, condition of interest, and contrast analyses suggest that the task performance strategy in MD participants maintains stronger connections between reward-related, self-referential, and cognitive processes compared to HC participants. This hyperconnectivity among reward, self-referential, and cognitive networks is a recognized feature of addiction [74, 75], corroborated by a recent meta-analysis of resting-state FC in nearly 2000 individuals with various addictions [76]. Second, we found that MD participants had a higher probability of occurrence of the DMN during the MID task, whereas the visual network was significantly more likely to occur in HC participants.

While we observed differences between HC and MD participants in miFC and dynamics during the MID task, the cue reactivity task only showed significant differences in miFC between MD and HC participants. While weaker connectivity in people with sOUD vs HC is a consistent feature of resting state-fMRI data [77], miFC results did not meet our hypothesis of greater miFC from VMN regions; rather, the results showed MD participants had weaker miFC between the somatomotor network and nearly all other networks.

However, the results from the drug cues > neutral cues contrast aligned with our hypotheses that MD participants would show hyperconnectivity between the VMN and other networks. The reliable hyperactivity of the VMN in sOUD vs HC participants during drug cue presentation is thought to reflect the increased motivational, incentive salience of drug cues [3, 13, 14]. The similar patterns of stronger between-network miFC in both the MID and cue reactivity task suggest that motivational and affective processes may influence task strategy and performance to a greater degree in MD than HC. This accords with Dunlop et al.’s “urge to action” trajectory, which posits that disrupted VMN function drives dysfunction in the networks underpinning other processes affected by addiction (e.g., cognitive, attentional, etc) [4].

The results from the PLS require cautious interpretation, given that the extent to which the latent variable explained the covariance between molecular predictors and functional differences between MD and HC participants did not survive spin tests. Therefore, the multivariate mapping between molecular properties and differences in connectivity may be driven by spatial autocorrelation [29, 78]. Spatial autocorrelation refers to the orgasational property of the brain where regions in close proximity share similar functions compared to more physically distant regions [79]. The influence of spatial autocorrelation on the PLS results is likely driven by the tight structure-function relationships between subcortical regions to processes engaged by both tasks. This rationale is supported when considering that the MOR availability was more important than the D2DR availability in the latent variable’s ability to explain the spatial relationship between molecular predictors and functional responses. MOR receptors are disproportionally more available in the subcortical VMN regions compared to other regions [32], and VMN regions with high MOR availability are also where edges with stronger miFC in MD vs HC participants were concentrated. The VMN was the network with the greatest contribution to the predictor score of the primary latent variable, and the only significant relationship between a molecular predictor and functional response was to the difference in eigenvector centrality during the MID task. In summary, the PLS analysis did not shed insight as to how molecular properties contributed to widespread, spatially distributed differences in function between MD and HC participants. Rather, it provided preliminary mechanistic evidence that the effects of sOUD operate on processes governed by tight structure-function relationships.

One limitation of this work is the small sample size, given that false positive results and inflated effect sizes are more common in samples <1000 [80]. Our use of task fMRI and multimodal analyses improves the reliability of results, and future work can utilise large neuroimaging repositories (e.g., the ENIGMA addiction working group) to evaluate the replicability of our findings. Large neuroimaging datasets are also better able to account for the variability introduced by demographic factors [81]. The significant between-group difference in smoking status, education, and psychiatric history reported in this work is not unusual for research in people with sOUD [82], but may have influenced the neuroimaging results. However, despite not achieving parity in representation of men and women per group, there was not a significant between-groups difference in the number of women per group, in line with recommendations from a recent review of clinical challenges in fMRI OUD research [83].

Another limitation of this work is the use of group-averaged PET images derived from healthy controls in our PLS. The obstacles to collect large datasets from clinical populations in fMRI studies [80] are further exacerbated in PET research, especially when investigating the role of multiple receptors on brain function. Moreover, it is important to note that the observed relationships between receptor availability and functional responses may reflect shared circuit-level anatomical organisation rather than direct molecular effects on neural function. The colocalisation of high receptor densities and strong functional responses within subcortical reward circuits could arise from common anatomical constraints rather than causal neurochemical mechanisms. Disentangling anatomical colocalisation from true molecular-functional relationships would require complementary methodological approaches, such as pharmacological manipulations or individual difference analyses that can better control for underlying anatomical organisation. Our work offers a starting position from which future PET-fMRI research can further characterise the relationship between receptor availability and brain function.

There are three strengths offered by our study design and analysis. First, FC is typically conducted with resting state data. However, task fMRI studies typically are higher powered than resting state studies, as the engaging nature of tasks reduces participant movement and sleepiness, thus increasing the SNR [84]. Second, our use of tasks with both non-drug and drug-related stimuli facilitated an assessment of whether the differences in miFC between HC and MD participants are generalisable, or context-dependent. Identifying differences in HC vs MD participants that generalise across tasks provides actionable targets for therapeutic interventions targeting dysregulated neural processes [85]. Future research is necessary to quantify the replicability and broader generalisability of these findings within other task conditions and addiction populations (e.g., people with sOUD who are abstinent). Third, our use of miFC provides a richer assessment of the relationship among functional networks a conventional whole brain voxelwise analysis. This is because miFC captures the strength of nonlinear relationships, providing a more concrete assessment of interregional, nonlinear relationships as compared to measures of coactivation [16, 39].

## Conclusions

These findings reveal distinct patterns of neural network interactions in individuals with sOUD. Across both tasks MD participants show generally weaker miFC than HC participants. During the MID task we show more robust VMN, DMN, and control network connectivity and dynamics in MD vs HC participants, as well as a high spatial correspondence between areas with high MOR and D2DR availability and functional differences between groups. Our results provide a foundation for future research to further characterise the relationship between the neural and behavioural correlates of sOUD, as well as investigate whether neuromodulatory interventions of the neural correlates of sOUD attenuate dysfunctional network connectivity and dynamics.

## Supplementary information

Supplementary Materials

Supp Mat Table 1

Supp Mat Table 2

Supp Mat Table 3

Supp Mat Table 4

Supp Mat Table 5

Supp Mat Table 6

Supp Mat Table 7

Supp Mat Table 8

Supp Mat Table 9

Supp Mat Table 10

Supp Mat Table 11

Supp Mat Table 12

Supp Mat Table 13

Supp Mat Table 14

Supp Mat Table 15

## Data Availability

The extracted timeseries used as inputs to compute miFC and brain state dynamics, make figures, and conduct statistical analysis described in this paper is available at https://github.com/daniellekurtin/miFC-dFC-PET-4-OUD.

## Code availability

The code to compute miFC and brain state dynamics, make figures, and conduct statistical analysis described in this paper is available at https://github.com/daniellekurtin/miFC-dFC-PET-4-OUD.
