# Supplementary material for: Task-related differences in network connectivity and dynamics in people with severe opioid use disorder compared with healthy controls
Source: Transl Psychiatry. 2026 Feb 3;16:111. doi: 10.1038/s41398-026-03845-6 (PMC12923702; doi:10.1038/s41398-026-03845-6)
Supplement: Supplementary file 1 — Supplementary Materials [file 41398_2026_3845_MOESM1_ESM.docx]

# SUPPLEMENTARY MATERIALS

**Task description:**

Our implementation of the cue reactivity task is described elsewhere^1^. Participants performed two runs of the cue reactivity paradigm. Each run utilised a fixed-order presentation of blocks of neutral cues, drug-related cues, and rest. There were 5 blocks neutral cues and 5 blocks of drug-related cues. The first block always consisted of neutral cues, followed by a block of drug-related cues, and then a rest block. The rest block presented a black screen with a fixation cross for 15 seconds. Each non-rest block contained 6 images/trials with a duration of 5 seconds, spaced apart with a jittered inter-trial interval ranging between 200-500 milliseconds. The hue, contrast, brightness, complexity, and content of images were controlled across neutral vs drug-related cues. People with opioid use disorder (OUD) attending a local treatment service participated in a focus group to provide feedback on drug cue content. Each run lasted 8 minutes and 20 seconds, and the second run utilised the same images as the first run. However, the second run shuffled the order of blocks and images within each block, while maintaining pattern of starting with a block of neutral cues, followed by a block of drug-related cues, then rest.

We used one run of a previously published version of the monetary incentive delay (MID) task^2^ that modified the original MID task^3^. The MID task contained 18 win, 6 loss, and 18 neutral trials. At the start of each trial, a symbol would appear indicating the type of trial (e.g., win, loss, or neutral). The disappearance of the symbol began an “anticipation” period of 2, 3, or 4 seconds (there were equal numbers of each duration balanced across the trial types). After the anticipation period, the target stimulus appeared for a brief duration. Participants were instructed to press a button as quickly as possible when they saw the target stimulus. Participants could win money during win trials by pressing the button fast enough (i.e., while the target stimulus was present) during win trials; alternatively, participants lost money during loss trials if they did not press the button fast enough. The starting duration for win and neutral trials was 280 milliseconds, and the starting duration for loss trials was 240 milliseconds. The duration of the target stimulus was changed for each trial type by an adaptive algorithm. The algorithm raised/lowered the target stimulus duration by 10 milliseconds if participants succeeded/missed a trial until a ceiling/floor of 300/150 milliseconds was reached, respectively. Then, 0.5 seconds after the disappearance of the target stimulus disappeared, participants were notified whether they were successful, and were shown their total winnings for 2 seconds. This completed the trial, after which an inter-trial interval of 2.4, 3.4, or 4.4 seconds occurred before the next trial began. The inter-trial interval durations were presented in equal numbers for each trial type. The task was designed for participants to win on 66% of the win trials and receive £5 in task winnings (completing the task with a perfect score would result in total winnings of £9).

**Power calculation**

This work is part of the larger Neural Correlates of Opiate Reward and Emotion (NCORE) study^1^. An a-priori power analysis was conducted to determine the sample size necessary to detect changes in the magnitude of the BOLD signal during the MID task due to pharmacological intervention. Therefore, our a-priori power calculation is neither suitable to infer whether our sample size was powered to detect differences in connectivity between HC vs MD participants, nor whether the partial least squared (PLS)-derived latent variables sufficiently captured the relationship between molecular properties and differences in connectivity metrics between HC vs MD participants. However, we estimated the post-hoc power to detect effects from PLS analysis based on the work by Andreella and colleagues^4^. With our total sample size of n=47, use of two latent variables and a nonparametric correlation-based test statistic, we were sufficiently powered (>0.75) to detect small-to-moderate effects.

To inform our post-hoc power to detect effects of group on connectivity with the sample size in this work, we utilised the approach by which the NCORE study determined the effect size used in the a-priori power calculation.

The a-priori power analysis for the NCORE study utilised the effect size of changes in the magnitude of the BOLD signal during the MID task from our previous ICCAM (Imperial College Cambridge Manchester) study^5^. While the primary outcomes for the ICCAM study also centred on changes in the magnitude of the BOLD signal, in a secondary connectivity analysis they showed there were 153 edges with significantly weaker connectivity in addiction vs HC populations^6^ during the MID task. While this does not provide a single effect size we can utilise in a formal post-hoc power analysis, we want to highlight how our study employs different methods to increase the power of our ability to detect differences in connectivity between MD and HC.

First, we utilised mutual information as our measure of connectivity (miFC), whereas McGonigle et al., employed a correlation-based approach. MiFC has been shown to be more sensitive than correlation-based metrics at detecting interregional relationships in healthy controls^7^, and better at capturing behaviourally relevant measures of brain function in clinical populations^8^. Therefore, the greater sensitivity of miFC vs correlation-based connectivity lends higher power to our analysis as to whether there are group differences in miFC.

Second, while Nestor et al used T-tests to assess between-groups differences in connectivity, we employed permutation tests. A direct comparison of the power obtained from parametric vs permutation tests of differences in connectivity showed permutation tests are consistently more sensitive and better powered, even with small sample sizes^9^.

In summary, we acknowledge that larger sample sizes consistently yield better power for detecting effects in whole-brain analysis, it was not possible to acquire a larger sample size for this project, given the patient population. However, we address the limitations of a smaller sample size by assessing the quality of the data itself (e.g., quality assessment metrics such as temporal signal to noise ratio (tSNR) and utilising connectivity and statistical methods that increase the power to detect effects.

**MRI acquisition**

MRI data was acquired over the course of an hour, with a 3T Siemens Magnetom Verio at the Clinical Imaging Facility at Imperial College London as previously reported^1^. Scanning sequences began with a T1-weighted MPRAGE (TR=2300 ms, TE=2.98 ms, TI=900 ms, flip angle=9°, FOV=256 mm, voxel size=1mm^3^), which was used as high-resolution anatomical images. The next four sequences were functional, beginning with a resting state sequence followed by the MID, evocative Images, and cue reactivity tasks. Only the functional data from the MID and cue reactivity sequences are analysed in this work. Functional images were acquired with a T2*-weighted gradient echo EPI sequence (TR=1500 ms, TE=30 ms, flip angle=62°, FOV=192 mm, voxel size=3mm^3^, 54 slices, 322 volumes) with in-plane (GRAPPA) acceleration factor of 2 and a multiband acceleration factor of 2. **Quality assessment**

Quality assessment was performed on the fMRI data both before and after preprocessing. Before preprocessing, head motion was estimated using FSL motion outliers through framewise displacement (FD) using *fsl_motion_outliers*^10^, and the number of noise components identified by ICA AROMA for each subject. After preprocessing, the global temporal signal to noise ratio (tSNR) was extracted as a metric of the stability and quality of the signal over time. TSNR was calculated by dividing the mean signal intensity across all voxels over time by the standard deviation of the voxel time course^11,12^. TSNR’s measurement of the temporal quality of the data is relevant for the miFC and brain dynamics analysis; however, the baseline activation levels inherent in task fMRI make tSNR a less robust measure of image quality^12^. Given our TR=1.5s, and 292 or 322 volumes for the MID and Cue Reactivity tasks, we require a tSNR>10 to detect an effect size of 5% with a p-value<0.05^11^.

**Leading Eigenvector Dynamic Analyses (LEiDA): Methods**

The initial steps of LEiDA, from demeaning to construction of the phase-based connectivity matrix, are shared by HBSD. However, LEiDA then extracts the leading eigenvector from each matrix, and performs k-means clustering on all leading eigenvectors. The centroid of each cluster comprises a brain state, and each timepoint from the leading eigenvectors in that cluster are assigned a label corresponding to that state. While the data derived topology of LEiDA’s states is informative, the interpretation of the functions it governs is often grounded by the relationship of its topology to a resting state network. By defining states as functional networks, HomeBrew State Dynamics (HBSD) streamlines the interpretation of how dynamical properties relate to functional processes. Moreover, the predefined number of states utilised in HBSD overcomes challenges inherent in k-selection approaches (i.e., choosing the number of states). A value of k is typically as the one with the best cluster performance metrics or that shows the most significant difference in metrics of state dynamics between groups. Cluster performance metrics are not well suited to fMRI data^13^, and reporting results based on a single value of k may not capture robust trends in state dynamics across several clustering solutions. We have previously overcome this challenge by reporting whether between-group differences in state dynamics are consistent across many values of k^14^. However, we introduce HBSD to both streamline the interpretation and results of state dynamics analysis.

**Leading Eigenvector Dynamic Analysis: Results**

Here we first present the results as to whether there are significant group differences in metrics of brain state dynamics between HC and MD participants for the MID and cue reactivity tasks, respectively. The results from each task will be compared to those reported in the main text.

Out of the 119 states identified across all values of K, there were 10 that showed a significant group difference in state lifetime and probability (Supplementary Tables 1-2, respectively). Five states showed differences in both state lifetime and probability, and there were 2 and 3 states with significant differences in either state lifetime or probability, respectively. States with significantly higher probability and/or lifetime of occurrence in HC vs MD participants tended to include regions within the visual cortex. States with significantly higher probability and/or lifetime of occurrence in MD vs HC participants tended to include regions in the DMN. This pattern of results aligns with the significant differences in DMN and visual network state dynamics between HC and MD participants observed in the main text. However, the relationship between these results should be interpreted with caution, since the effects of group on state dynamics does not replicate consistently across values of K, and that most significant differences in state lifetime and probability between groups occur when K>8. We have previously described that results with consistent replication over different values of K are more robust, and less likely attributed to the higher variance in state dynamics observed in higher values of K^14^.

The variability of results across values of K extend to the results observed for the difference in information theoretic metrics (Supplementary Materials Table 3). There were three values of K where MD participants had significantly higher BDMC, 2^nd^, 3^rd^, and 4^th^ order Transition Entropy than HC participants. It would be difficult to consider replication across 3 out of 14 values of K robust, especially since most results occurred when K=8 or higher, and the results per information metric did not replicate consistently across values of K. In summary, while the significant differences in metrics of state dynamics between HC and MD participants align with those reported in the main text, the weak reliability of these results warrant caution in their interpretation.

Significant differences in metrics of state dynamics between HC and MD participants during the cue reactivity task follow a similar pattern to those during the MID task. For example, the significant group differences in state lifetime, probability, and information theoretic metrics mostly occur when K>8 (Supplementary Tables 4-6, respectively). Interpretation of these results is difficult, given both the questionable reliability of the LEiDA-derived results, and that there were no significant group differences in metrics of state dynamics observed in the main text. Further exploration of the potential relationship between the state topology observed in LEiDA-derived states and the differences in miFC observed between HC and MD participants is beyond the scope of this work but is encouraged in future work investigating how differences in state topology and dynamics between HC and MD participants.

**Supplementary Table 1:** Results from permutation tests evaluating the effect of group on state lifetime during the MID task for each state in value of K. Results are presented as p-value, T-stat, and Cohen’s D. Results that are significant after max-T familywise error correction are highlighted.

**Supplementary Table 2:** Results from permutation tests evaluating the effect of group on state probability during the MID task for each state in value of K. Results are presented as p-value, T-stat, and Cohen’s D. Results that are significant after max-T familywise error correction are highlighted.

**Supplementary Table 3:** Results from permutation tests evaluating the effect of group on information theoretic metrics of state dynamics during the MID task for each value of K. Results are presented as p-value, T-stat, and Cohen’s D. Results that are significant after max-T familywise error correction are highlighted. Abbreviations are as follows: Transition Entropy (TE); Block Decomposition Methods of Complexity (BDMC); Lempel Ziv Complexity (LZC).

**Supplementary Table 4:** Results from permutation tests evaluating the effect of group on state lifetime during the cue reactivity task for each state in value of K. Results are presented as p-value, T-stat, and Cohen’s D. Results that are significant after max-T familywise error correction are highlighted.

**Supplementary Table 5:** Results from permutation tests evaluating the effect of group on state probability during the Cue Reactivity task for each state in value of K. Results are presented as p-value, T-stat, and Cohen’s D. Results that are significant after max-T familywise error correction are highlighted.

**Supplementary Table 6:** Results from permutation tests evaluating the effect of group on information theoretic metrics of state dynamics during the cue reactivity task for each value of K. Results are presented as p-value, T-stat, and Cohen’s D. Results that are significant after max-T familywise error correction are highlighted. Abbreviations are as follows: Transition Entropy (TE); Block Decomposition Methods of Complexity (BDMC); Lempel Ziv Complexity (LZC).

**Supplementary Table 7: Clinical and demographic characteristics of the MD and HC participants.**

Data shown as mean ± std or median with (range). We employed Chi-Squared test statistical comparisons for differences in demographics between HC and MD participants in nominal data, or **Fisher's Exact Test when expected cell counts were low. If Kolgov-Smirnov tests showed data did not meet assumptions for normal distributions, we used** the Mann-Whitney U test; otherwise, unpaired t-tests were used for comparisons between parametric data. Smoking status includes those who were vapers at the time of the study. Abbreviations: *DSM-5; Diagnostic and Statistical Manual of Mental Disorders 5^TH^ Edition.

|  | **HC** | **MD** | **Group Comparisons** |
| --- | --- | --- | --- |
| **n** | 22 | 25 |  |
| **Age (years)** | 46.9±10.9 (30-65) | 43.5±10.0 (19-60) | t(46)=-1.12, p=0.30 |
| **Females, n (%)** | 4 (9.1%) | 6 (23.1%) | χ^2^(1, 48)=0.70, p=0.40 |
| **Years of education** | 15.6±2.34 (11-20) | 13.1±2.56 (9-20) | t(46)=-3.60, p<0.001 |
| **Caucasian, n (%)** | 18 (81.8%) | 16 (61.5%) | χ^2^(1,48)=2.37, p=0.12 |
| **Screening methadone dose (mg)** |  | 33.0±16.8 (2-85) |  |
| **Age of first heroin use** |  | 24.5±8.0 (11-49) |  |
| **Total years on methadone** |  | 8.2±6.8 (0.08-30) |  |
| ***DSM-5 Peak Crack use** |  | 4.4±4.9 (0-11) |  |
| **Smoking status, n (%)** | 22 (81.5%) | 49 (98.0%) | **OR=11.14, p=0.02** |
| **Current Psychiatric Diagnosis, n (%)** | 0 (0%) | 9 (34.6%) | χ^2^(1,48)=9.37, p=0.002 |

**Supplementary Table 8:** Edges with a significant difference in miFC between HC and MD participants after max-T familywise correction for multiple comparisons during the MID task.

**Supplementary Table 9:** Edges with a significant difference in miFC between HC and MD participants after max-T familywise correction for multiple comparisons during reward anticipation periods from the MID task.

**Supplementary Table 10:** Edges with a significant difference in miFC between HC and MD participants after max-T familywise correction for multiple comparisons during reward anticipation periods vs neutral anticipation periods from the MID task.

**Supplementary Table 11:** Edges with a significant difference in miFC between HC and MD participants after max-T familywise correction for multiple comparisons during the cue reactivity task.

**Supplementary Table 12:** Edges with a significant difference in miFC between HC and MD participants after max-T familywise correction for multiple comparisons during drug-stimuli cue blocks from the cue reactivity task.

**Supplementary Table 13:** Edges with a significant difference in miFC between HC and MD participants after max-T familywise correction for multiple comparisons during drug-stimuli cue blocks vs neutral blocks from the cue reactivity task.

**Supplementary Table 14:** The effect of group on state lifetime and probability during the MID and cue reactivity tasks.

**Supplementary Table 15:** The effect of group on information theoretic metrics of state dynamics during the MID and cue reactivity tasks.

**REFERENCES:**

1. Fonville, L. *et al.* Functional evaluation of NK1 antagonism on cue reactivity in opiate dependence; An fMRI study. *Drug and Alcohol Dependence* **221**, 108564 (2021).

2. McGonigle, J. *et al.* The ICCAM platform study: An experimental medicine platform for evaluating new drugs for relapse prevention in addiction. Part B: fMRI description. *J Psychopharmacol* **31**, 3–16 (2017).

3. Knutson, B., Adams, C. M., Fong, G. W. & Hommer, D. Anticipation of increasing monetary reward selectively recruits nucleus accumbens. *The Journal of Neuroscience* **21**, RC159–RC159 (2001).

4. Andreella, A., Fino, L., Scarpa, B. & Stocchero, M. Towards a power analysis for PLS-based methods. Preprint at https://doi.org/10.48550/arXiv.2403.10289 (2024).

5. Paterson, L. M. *et al.* The Imperial College Cambridge Manchester (ICCAM) platform study: An experimental medicine platform for evaluating new drugs for relapse prevention in addiction. Part A: Study description. *J Psychopharmacol* **29**, 943–960 (2015).

6. Nestor, L. J. *et al.* Disturbances across whole brain networks during reward anticipation in an abstinent addiction population. *NeuroImage: Clinical* **27**, 102297 (2020).

7. Hlinka, J., Paluš, M., Vejmelka, M., Mantini, D. & Corbetta, M. Functional connectivity in resting-state fMRI: Is linear correlation sufficient? *NeuroImage* **54**, 2218–2225 (2011).

8. Zhang, W., Muravina, V., Azencott, R., Chu, Z. D. & Paldino, M. J. Mutual Information Better Quantifies Brain Network Architecture in Children with Epilepsy. *Comput Math Methods Med* **2018**, 6142898 (2018).

9. Goulden, N. *et al.* A comparison of permutation and parametric testing for between group effective connectivity differences using DCM. *NeuroImage* **50**, 509–515 (2010).

10. Power, J. D., Barnes, K. A., Snyder, A. Z., Schlaggar, B. L. & Petersen, S. E. Spurious but systematic correlations in functional connectivity MRI networks arise from subject motion. *NeuroImage* **59**, 2142–2154 (2012).

11. Murphy, K., Bodurka, J. & Bandettini, P. A. How long to scan? The relationship between fMRI temporal signal to noise ratio and necessary scan duration. *NeuroImage* **34**, 565–574 (2007).

12. Welvaert, M. & Rosseel, Y. On the Definition of Signal-To-Noise Ratio and Contrast-To-Noise Ratio for fMRI Data. *PLoS One* **8**, e77089 (2013).

13. Kurtin, D. L. Context-dependent reconfigurations in brain state topology and dynamics. (University of Surrey, 2023). doi:10.15126/thesis.900686.

14. Kurtin, D. L., Scott, G., Hebron, H., Skeldon, A. C. & Violante, I. R. Task-based differences in brain state dynamics and their relation to cognitive ability. *NeuroImage* **271**, 119945 (2023).
